# Ipsilateral EEG mu rhythm reflects the excitability of uncrossed pathways projecting to shoulder muscles

**DOI:** 10.1186/s12984-017-0294-2

**Published:** 2017-08-25

**Authors:** Keita Hasegawa, Shoko Kasuga, Kenichi Takasaki, Katsuhiro Mizuno, Meigen Liu, Junichi Ushiba

**Affiliations:** 1 Graduate School of Science and Technology, Keio University, 3-14-1, Hiyoshi, Kohoku-ku, Yokohama, Kanagawa 223-8522 Japan; 2 Keio Institute of Pure and Applied Sciences (KiPAS), 3-14-1, Hiyoshi, Kohoku-ku, Yokohama, Kanagawa Japan; 3 Department of Rehabilitation Medicine, Keio University School of Medicine, 35, Shinanomachi, Shinjuku, Tokyo, 160-8582 Japan; 4 Faculty of Science and Technology, Keio University, 3-14-1, Hiyoshi, Kohoku-ku, Yokohama, Kanagawa 223-8522 Japan

**Keywords:** Electroencephalography, Brain-computer interface, Event-related desynchronization, Stroke rehabilitation

## Abstract

**Background:**

Motor planning, imagery or execution is associated with event-related desynchronization (ERD) of mu rhythm oscillations (8-13 Hz) recordable over sensorimotor areas using electroencephalography (EEG). It was shown that motor imagery involving distal muscles, e.g. finger movements, results in contralateral ERD correlating with increased excitability of the contralateral corticospinal tract (c-CST). Following the rationale that purposefully increasing c-CST excitability might facilitate motor recovery after stroke, ERD recently became an attractive target for brain-computer interface (BCI)-based neurorehabilitation training. It was unclear, however, whether ERD would also reflect excitability of the ipsilateral corticospinal tract (i-CST) that mainly innervates proximal muscles involved in e.g. shoulder movements. Such knowledge would be important to optimize and extend ERD-based BCI neurorehabilitation protocols, e.g. to restore shoulder movements after stroke. Here we used single-pulse transcranial magnetic stimulation (TMS) targeting the ipsilateral primary motor cortex to elicit motor evoked potentials (MEPs) of the trapezius muscle. To assess whether ERD reflects excitability of the i-CST, a correlation analysis between between MEP amplitudes and ipsilateral ERD was performed.

**Methods:**

Experiment 1 consisted of a motor execution task during which 10 healthy volunteers performed elevations of the shoulder girdle or finger pinching while a 128-channel EEG was recorded. Experiment 2 consisted of a motor imagery task during which 16 healthy volunteers imagined shoulder girdle elevations or finger pinching while an EEG was recorded; the participants simultaneously received randomly timed, single-pulse TMS to the ipsilateral primary motor cortex. The spatial pattern and amplitude of ERD and the amplitude of the agonist muscle’s TMS-induced MEPs were analyzed.

**Results:**

ERDs occurred bilaterally during both execution and imagery of shoulder girdle elevations, but were lateralized to the contralateral hemisphere during finger pinching. We found that trapezius MEPs increased during motor imagery of shoulder elevations and correlated with ipsilateral ERD amplitudes.

**Conclusions:**

Ipsilateral ERD during execution and imagery of shoulder girdle elevations appears to reflect the excitability of uncrossed pathways projecting to the shoulder muscles. As such, ipsilateral ERD could be used for neurofeedback training of shoulder movement, aiming at reanimation of the i-CST.

## Background

Sensorimotor rhythm (SMR) is a feature of scalp electroencephalography (EEG) of the temporal region and is interpreted as a signature of thalamocortical activity [1]. Voluntary decrease of the SMR, named Event-Related Desynchronization (ERD), can be induced by unilateral hand motor execution and is often observed in the sensorimotor area (C3, C4, and Cz of the international 10-20 system) of the contralateral hemisphere [2–4]. ERD is accompanied by the excitation of corticomuscular pathways [5] through disinhibition of GABAergic interneurons in the primary motor cortex (M1) [1] and potentiation of spinal motor neuron pools [6]. ERD also occurs in the absence of physical movement, and a neural substrate for learning ERD generation is shared with that for the acquisition of physical motor skills [7]. Contralateral ERD-based neurofeedback training has been, therefore, used for reanimating corticomuscular functional integrity to restore hand/finger function in severe hemiplegia due to stroke [8, 9].

Unlike hand motor recovery occurring through the disinhibition of contralateral corticomuscular pathways [10, 11], functional recovery of axial or shoulder muscles from stroke hemiplegia is promoted by unmasking the ipsilateral pathway to the paretic hand [12–15]. Thus, neurofeedback using ipsilateral ERD may be conceptually useful for functional maturation of ipsilateral corticomuscular pathways, aiming for proximal muscle motor recovery.

The major premise for this idea is that ipsilateral ERD during shoulder motor imagery reflects ipsilateral corticospinal excitability. Contralateral ERD during hand motor imagery is known to be associated with contralateral corticospinal excitability [1, 6], but nothing is assured concerning the ipsilateral proximal projection. The neuroanatomical properties of cortical projections to shoulder muscles are different from those to distal muscles [16–20], and intraspinal circuitries also differ [21].

We, therefore, tested the association of ipsilateral ERD with ipsilateral corticomuscular pathways during shoulder motor imagery by using single-pulse TMS. The association between the amplitudes of the motor evoked potentials (MEP) elicited by TMS and ERD prior to TMS was analyzed.

## Methods

### Ethics statement

This study was conducted according to the Declaration of Helsinki. The experimental procedures were approved by the ethical committee of the Faculty of Science and Technology, Keio University (#25-32). Written, informed consent was obtained from all participants prior to the experiments. We also obtained written consent prior to the experiments to publish data from all participants.

### Participants

Ten individuals participated in experiment 1 (1 woman and 9 men, ages 22–28 years), and 16 individuals participated in experiment 2 (5 women and 11 men, ages 22–29 years). The sample size was different between experiments because of following reasons. The purpose of the experiment 1 is to assess the spatial distribution of ERD. With this purpose, we recruited 10 participants, since a sample size for ERD observation in motor execution task is empirically estimated around 9-14 [2, 4]. On the contrary, in the experiment 2, we aimed to see the association between ERD and MEP. The required sample size for ERD-MEP studies is estimated around 10-20 [1, 22] with consideration of ERD/MEP fluctuation in nature [1], thus we recruited 16 participants in this experiment.

All of the participants were neurologically healthy and naïve to the purpose of the experiment. Participants were recruited from a homogeneous population (i.e., healthy, untrained young adults) to minimize the potential influence of factors such as neurological disorders [23–25], learning experience/habitude [26, 27], and aging [28, 29] on corticomuscular function.

In experiment 2, two participants were excluded from the MEP analysis in the shoulder motor imagery task, and one participant was excluded from the MEP analysis in the finger motor imagery task, because MEPs were not evoked in the target muscle.

### Experimental procedure

We first conducted experiment 1 to ensure that the ipsilateral ERD to the executed side of shoulder movement could be observed. Since experiment 1 successfully demonstrated the feasibility of the concept described above, next we conducted experiment 2 to see the correlation between ipsilateral ERD during shoulder motor imagery and MEP.

### Experiment 1

**Fig. 1:**
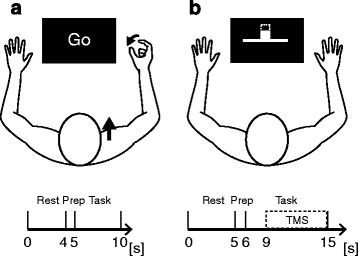
A schematic representation of the experimental protocols. Rest: rest period, Prep: preparation period, Task: task period (motor execution, motor imagery, or relax). **a** Experiment 1 in which participants performed movement execution (finger pinching or elevation of the shoulder girdle) during the task period. When the word ‘Go’ was presented on the computer monitor, participants executed a movement that was specified during the preparation period. **b** Experiment 2 in which participants performed motor imagery (finger pinching or elevation of the shoulder girdle) or remained relaxed without any imagery during the task period. When participants performed imagery of movements, the magnitude of ERD was fed-back on the computer monitor

Participants were seated in a firm-backed chair with their forehead and arms supported on a headrest and an armrest. A monitor was placed 60–90 cm in front of the participants. The experiment consisted of 100 trials of a motor execution task. Each trial started with a rest period indicated by the presentation of the word “Rest” on the monitor for 4 s. After the rest period, the participants were prepared for the movement required to be performed in the upcoming motor execution period by displaying the word “Finger” or “Shoulder” on the monitor for 1 s during the preparation period. Movement types were randomized across trials. When the word “Go” was displayed, the participants performed the motor execution task for 5 s. Based on the previous ERD observation studies [30, 31], 5 s of the task period was used in this experiment. During the motor execution period, participants were asked to either form a pinch grip between the right index finger and the right thumb or elevate the right shoulder girdle without any other movement of the upper-limb extremity at a frequency of 1 Hz (one movement per second). Pacing of the movements was practiced using a metronome prior to the experiment, and no external trigger was provided during the experiment. A schematic representation of the experimental procedure is represented in Fig. 1a.

### Experiment 2

The experimental apparatus was the same as in experiment 1; however, in experiment 2, participants were instructed to keep their arms and hands relaxed during the task. The experiment consisted of two blocks of 90 trials. One block tested the condition involving right finger motor imagery with left-sided (contralateral) TMS, and another block tested the condition involving right shoulder motor imagery with right-sided (ipsilateral) TMS. Two blocks were performed on the same day, and were performed 10 min apart. The order of the blocks was randomized for each participant. Each trial started with a rest period indicated by the presentation of the word “Rest” on the monitor for 5 s. After the rest period, the participants were prepared for the task with the word “Imagery” or “Relax” displayed for 1 s during the preparation period. Sixty trials of the motor imagery task were performed to investigate the relationship between cortical excitability and ERD, and 30 trials of the relaxation task (i.e., without any motor imagery) were used to evaluate resting cortical excitability. When the word “Go” was displayed, participants executed the indicated task for 9 s. In order to avoid anticipatory changes of the participant’s brain activity, we employed the task period of 9 s in this experiment, and shuffled TMS timing ranged 3-9 s. Since we conducted the experiments 1 and 2 separately for respective different purposes, the task period was each arranged.

During the motor imagery period, the participants were asked to imagine with maximal effort either adducting their right index finger or elevating their right shoulder girdle without any movement of the upper-limb extremity. They were instructed to imagine the movements occurring at a frequency of 0.5-1 Hz. Pacing of the movements was practiced using a metronome prior to the experiment, and no external trigger was provided during the experiment. Although basically a frequency of motor imagery was same as experiment 1 (1 Hz), we instructed some of the participants who claimed the difficulty of the task to employ slower frequency of motor imagery up to 0.5 Hz, in order to manage task compliance. We monitored the electromyography (EMG) over the trapezius during the motor imagery task, and verbally instructed the participants to relax the muscle if any EMG signals were detected. While the participants performed the motor imagery task, the amplitude of ERD was displayed on the monitor as feedback. The ERD amplitude was represented as a moving ball on the screen, and the participants were encouraged to keep the ball moving upward. Visual feedback to encourage participants to generate a similar level of ERD across trials and/or participants was given with regards to the ERD calculated in the corresponding hemisphere. TMS was applied with random timing for a duration of 3–9 s during this period (see *TMS protocol*). A schematic representation of the experimental procedure is represented in Fig. 1b.

### Data recording

#### EEG

In both experiment 1 and 2, EEG was recorded with 128 electrodes (Geodesic Sensor Nets; Electrical Geodesics Incorporated, Eugene, OR, USA). Electrode impedance was kept below 50 kΩ throughout the experiment. The EEG signals were amplified, band-pass filtered (5–70 Hz), notch filtered (50 Hz), and digitized at 250 Hz using Geodesic EEG System 400 (Electrical Geodesics Incorporated).

#### EMG

In experiment 2, EMG was recorded with Ag/AgCl electrodes (10 mm diameter). The cathode and anode electrodes were placed in a belly-tendon montage over the first dorsal interosseous (FDI) muscle for finger motor imagery and over the right trapezius muscle for shoulder motor imagery. EMG signals were band-pass filtered (3–70 Hz with second-order Butterworth), notch filtered (50 Hz), and digitized at 250 Hz using a Physio16 input box for the Geodesic EEG System 400 (Electrical Geodesics Incorporated).

#### TMS protocol

In experiment 2, TMS was performed using a Magstim 200 magnetic stimulator (Magstim, Whitland, UK) connected to an angulated (95°) double cone coil (outer diameter of each coil, 11 cm). The coil was optimally positioned to obtain MEP in the FDI or trapezius muscle with the lowest stimulus intensity. The position of the TMS coil was maintained using the Brainsight TMS navigation system (Rogue Research, Cardiff, UK), so that the coil remained within 3-mm radius of the initial position. The resting motor threshold (RMT) was defined as the minimum stimulator output intensity that evoked MEP amplitudes larger than 50 μV in 5 out of 10 trials with relaxed muscles. Single-pulse TMS was applied with an intensity of 120% of the RMT at a random time during the 3–9 s motor imagery period. We randomized the time of TMS application so that participants could not anticipate the stimulation.

### Data analysis

#### Offline ERD quantification

In experiment 1, event-related trials during motor execution were selected for offline data processing. The EEG signals were common average referenced [32] by subtracting the average signal recorded over all electrodes from each electrode at each time point. We used a CAR spatial filter for offline analysis, because we wanted to investigate spatial feature of ERD over the scalp. A Laplacian filter, which was used for the online ERD quantification described in the following section, fails to detect brain activities on some of the peripheral electrodes due to its algorithm, and thus have some difficulty for the topography analysis. Also, unevenly spaced electrode locations in this study may make difficulty of Laplacian filtering. Use of CAR in offline analysis is considered feasible since Laplacian and CAR are highly correlated [33], and both are best for high signal-to-noise ratio [34]. The mathematical theory in spatial filtering says that Laplacian is local, and CAR is global [35], supporting our idea on the selection of spatial filters depending on the purpose in on-line (local cortical activity feedback) and off-line (spatial/topographic analysis) analyses. Independent component analysis was then applied to remove electrooculogram or eyeblink artifact-related components. Trials were segmented into successive 1 s windows with 225 overlapping sample points, and the power spectral density in each segment was calculated with a Hamming window. ERD was expressed as the power decrease in relation to a 2 s reference interval (rest period) before the preparation period. The offline ERD in decibels (dB) was calculated for each time point with a resolution of 0.1 s and a frequency according to Eq. 1,

1 $$ ERD\left(f,t\right)=10{\mathrm{log}}_{10}\frac{A\left(f,t\right)}{R(f)}, $$

where, *A* is the power spectral density of the recorded EEG at a certain frequency *f* (Hz) at time *t* (s) with reference to the onset of motor execution or imagery, and *R* is the power spectral density in the 2–4 s rest period. We applied a dB conversion for offline ERD analysis [36], because it ensures that all time points, electrodes, conditions, and participants are on the same scale and, thus, comparable. In addition, the probability distribution of the alpha band power typically forms the log-normal distribution, but not the Gaussian distribution [37, 38]. Thus, the logarithmic transformation yields the alpha-band power distribution to be a Gaussian. The present study applied this processing for correcting the weights of ERD and ERS in calculation of LI. Some previous articles actually took this policy especially when signals take both ERD(−)/ERS(+) values [37, 39, 40]. As the logarithm is a monotonic transformation it preserves the neighborhood property of the data and hence does not affect any monotonic relationship between the explanatory variable and the ERD characteristic [37].

In the current study, we examined ERD in the alpha band (8–13 Hz) for offline ERD analysis. We excluded trials from the offline analysis in which abnormally large alpha oscillations at occipital areas or body-motion artifacts were observed.

#### Online ERD quantification

To provide online ERD amplitude feedback to the participants in experiment 2, we calculated the ERD amplitude at electrode C3 online. The EEG signals were re-referenced using a Laplacian algorithm [41], which takes the difference between the potential at a corresponding electrode and the mean potential of its six nearest-neighbor electrodes. The EEG data were segmented into successive, 250 sample-point (1 s) windows with 225 overlapping sample points, and a fast Fourier transformation with a Hanning window was applied to each segment. The ERD amplitude displayed on the monitor was updated during the motor imagery task every 100 ms. The online ERD was calculated as a percentage as follows:

2 $$ ERD\left(f,t\right)=\frac{R(f)-A\left(f,t\right)}{R(f)}\times 100\%, $$

In the current study, we examined ERD in the alpha band (8–13 Hz) for online ERD quantification and visual feedback.

#### Laterality index

Hemispheric dominance was evaluated with the laterality index (LI) as described in Eq. 3,

3 $$ LI=\frac{\left({ERD}_{left}-{ERD}_{right}\right)}{\left|{ERD}_{left}\right|+\left|{ERD}_{right}\right|}, $$

where, ERD_left_ is the ERD in the left hemisphere (contralateral to the target muscles), and ERD_right_ is the ERD calculated in the right hemisphere (ipsilateral to the target muscles). The ERD used here is the average ERD between 2 and 4 s in the task period. LI should take values between −1 and 1. Since, in our calculation (Eqs. 1 and 2), ERD took a negative value if EEG desynchronization was observed during the task period, −1 indicates that ERD was observed only in the left hemisphere, and 1 indicates that ERD was observed only in the right hemisphere. For calculation of the LI, a total of 72 out of 128 electrodes were divided into 8 regions of interest (Front Left, FL, 8 channels; Central Left, CL, 10 channels; Posterior Left, PL, 9 channels; Occipital Left, OL, 9 channels; Front Right, FR, 8 channels; Central Right, CR, 10 channels; Posterior Right, PR, 9 channels; Occipital Right, OR, 9 channels). In experiment 1, the regions of interest used for calculation of the LI were visually determined for each hemisphere. The reason why we applied a visual selection of LI was because i) visual inspection is an established method to evaluate EEG features such as artifacts [42, 43], time-frequency map [44], and cortical topography [45]; ii) it helps us to avoid detecting regions which is not physiologically plausible, when an ERD accidently took large values at these regions due to electrooculogram and other noises. Based on visual inspection, we selected the areas where eye-blink noise and contact to the TMS coil or headrest were smallest, and were closest to the left primary sensorimotor cortex. In experiment 2, that region was selected for calculation of LI in which the channel that showed the lowest correlation coefficient between ERD and MEP was present. The LI was calculated using the mean ERD amplitude across channels in the region.

#### MEP analyses

In experiment 2, individual EMG sweeps were assessed visually, and trials with artifacts were rejected. The artifact-free MEP amplitudes were then measured peak-to-peak and used for further analysis.

#### Statistics

A Wilcoxon signed-rank test was performed for comparisons between the LI in shoulder vs. finger motor execution (experiment 1), and in shoulder vs. finger motor imagery (experiment 2). A Mann-Whitney U test was performed for comparisons between ERD amplitude in finger motor execution vs. imagery and shoulder motor execution vs. imagery. A paired *t*-test was used for comparison between MEP during shoulder motor imagery and the rest period (experiment 2).

Correlations between MEP and ERD calculated from data 1 s before TMS application were calculated by Spearman’s rank correlation (experiment 2). Then, a one-sample *t*-test was performed to investigate if correlation coefficients were significantly biased toward positive or negative values at the across-participants level.

## Results

### ERD was observed in both hemispheres during execution of elevation of the shoulder girdle

**Fig. 2:**
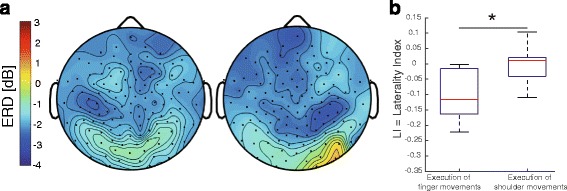
**a** Event-related desynchronization (ERD) topography during motor execution in a representative participant. Black dots indicate electroencephalogram (EEG) channel location. Left panel, finger motor execution; Right panel, shoulder motor execution. **b** Laterality index (LI) for finger and shoulder motor execution. An asterisk denotes statistical significance (*p* < 0.05)

The scalp topography of ERD during motor execution of right finger pinching and during elevation of the shoulder girdle in experiment 1 is shown in Fig. 2a. For right finger pinching, the topography qualitatively showed that a stronger ERD was observed on the left side than the right side. However, ERD was observed bilaterally for elevation of the right shoulder girdle. To quantify this topographic difference between finger pinching and elevation of the shoulder girdle, we calculated the LI of the ERD amplitude (see *Laterality Index* in the Methods). There was a significant difference in the LI (data shown as mean ± standard deviation) between elevation of the shoulder girdle and finger pinching (shoulder, 0.00 ± 0.07; finger, −0.10 ± 0.08; Wilcoxon signed-rank test, *p* < 0.05; Fig. 2b).

### ERD was observed in bilateral hemispheres during imagery of elevation of the shoulder girdle

**Fig. 3:**
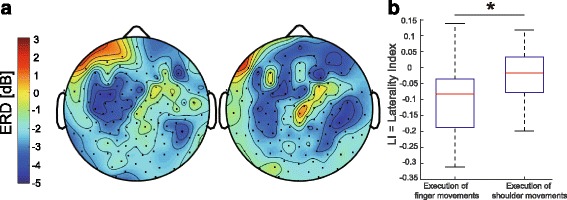
**a** Event-related desynchronization (ERD) topography during motor imagery in a representative participant; Black dots indicate electroencephalogram (EEG) channel location. *Left panel*, finger motor imagery; *Right panel*, shoulder motor imagery; **b** Laterality index (LI) for finger and shoulder motor imagery. An asterisk denotes statistical significance (*p* < 0.05)

The scalp topography of ERD during motor imagery of right finger pinching and elevation of the shoulder girdle in experiment 2 is shown in Fig. 3a. For right finger imagery, the topography qualitatively showed that stronger ERD was observed on the left side than the right side. On the other hand, ERD was observed bilaterally for right shoulder motor imagery. We quantified this difference by calculating the LI in the regions of interest (see *Laterality Index* in Methods). The regions of interest for finger imagery was same as shoulder imagery. We found that the LI was closer to zero (ERD in bilateral hemispheres) during shoulder motor imagery compared with finger motor imagery, where ERD was lateralized to the left/contralateral hemisphere. At the group level, the LI was also significantly larger (i.e., close to zero) in shoulder motor imagery (shoulder, −0.03 ± 0.10; finger, −0.10 ± 0.12; Wilcoxon signed-rank test, *p* < 0.05; Fig. 3b).

### ERD amplitude was not different between motor execution and imagery

We compared ERD amplitudes between execution and imagery of elevation of the shoulder girdle and finger pinching. ERD amplitudes were not different between conditions, but there was a trend for increased ERD amplitudes during execution when compared to imagery (execution, −3.17 ± 0.58 dB; imagery, −2.45 ± 0.87 dB; Mann-Whitney test, *p* = 0.08). For elevation of the shoulder girdle, there was no difference between execution and imagery (execution, −2.70 ± 0.96 dB; imagery, −2.55 ± 1.02 dB; Mann-Whitney test, *p* = 0.77).

### MEP in the shoulder muscles elicited by TMS was enlarged during the motor imagery task

**Fig. 4:**
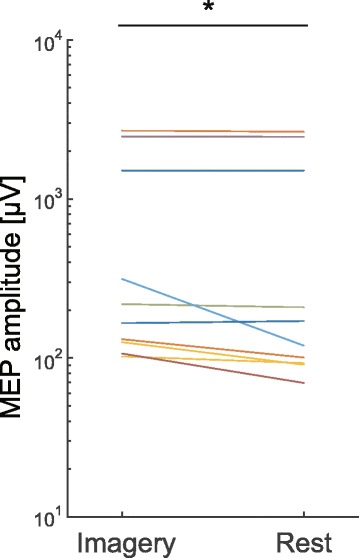
Motor-evoked potential (MEP) amplitude during motor imagery of elevation of the shoulder girdle and the rest period. Each line indicates data from an individual participant. An asterisk denotes statistical significance (*p* < 0.05)

MEP in the trapezius during imagery of elevation of the shoulder girdle and the rest period are shown in Fig. 4 and Table 1. There was a significant increase in MEP during motor imagery compared with the rest period (imagery, 608.7 ± 912.9 μV; rest, 576.2 ± 916.6 μV; *t*_13_ = 2.44, *p* < 0.05).

**Table 1:** MEP in the trapezius during imagery of elevation of the shoulder girdle and the rest period

Participant	MEP (Image) [μV]	MEP (Rest) [μV]
1	1508.4	1507.3
2	2691.1	2644.2
3	102.2	92.8
4	2470.1	2455.7
5	218.0	208.1
6	313.6	119.5
7	106.5	69.6
8	165.5	170.6
9	131.1	100.6
10	No Data	No Data
11	125.6	90.6
12	No Data	No Data
13	140.2	96.1
14	366.2	372.1
15	125.6	90.6
16	57.2	48.8

### MEP in the shoulder muscle and ERD in the ipsilateral hemisphere were correlated

**Fig. 5:**
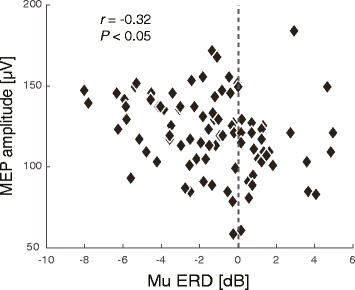
Relationship between event-related desynchronization (ERD) and motor-evoked potential (MEP) amplitude during shoulder motor imagery in a representative participant. Each diamond indicates a single trial

Figure 5 shows the correlation between MEP in the trapezius muscle and ERD in the ipsilateral hemisphere during motor imagery task of elevation of the shoulder girdle. The ERD amplitude represents the value calculated prior to 1 s of TMS. There were negative correlations in 12 out of the 14 participants (Table 2; significant in 6 out of 14 participants, *p* < 0.05). A one-sample *t*-test indicated that the correlations were also significantly negative at the across-participants level (*t*_13_ = −3.80, *p* < 0.01).

**Table 2:**
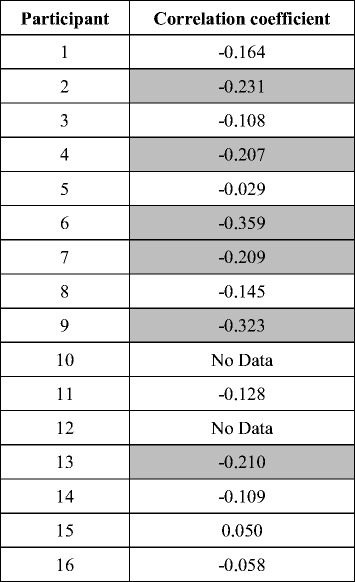
Correlations between amplitudes of MEP in the trapezius and ipsilateral ERD during imagery of shoulder movements. Shadowed values indicate significant correlation (*p* < 0.05)

### MEP in the finger muscles and ERD in the contralateral hemisphere were correlated

As a validation of our experimental procedure, we also investigated the correlation between MEP in the FDI and ERD in the contralateral hemisphere during motor imagery task of finger pinching. There were negative correlations in 14 of the 15 participants (Table 3; significant in 5 out of 15 participants, *p* < 0.05). A one-sample *t*-test indicated that the correlations were also significantly negative at the across-participants level (*t*_14_ = −4.10, *p* < 0.01).

**Table 3:**
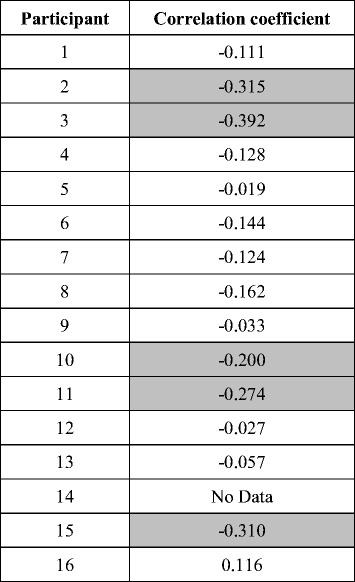
Correlations between amplitudes of MEP in the FDI and contralateral ERD during imagery of finger movements. Shadowed values indicate significant correlation (*p* < 0.05)

## Discussion

To determine whether ipsilateral ERD is associated with ipsilateral corticospinal pathways projecting to proximal muscles, the current study investigated the correlation between ipsilateral ERD during shoulder motor imagery and shoulder MEP elicited by single-pulse TMS.

We investigated the difference in ERD topography between movement execution in these muscles prior to the examination of ipsilateral corticospinal tract (i-CST) excitability during motor imagery to determine whether anatomical differences of corticospinal innervation between shoulder and finger muscles are reflected in a surface EEG feature. During the execution of elevation of the shoulder girdle, we found ERD in both hemispheres. However, during the execution of finger pinching, we found dominant ERD in the contralateral hemisphere, which is similar to that observed in some previous studies [46–49]. Anatomically, there are more ipsilateral (uncrossed) innervations of proximal muscles (including the trapezius) than there are of distal muscles [16–20]. ERD in the ipsilateral hemisphere may reflect desynchronized activity of neurons projecting through uncrossed pathways to the trapezius.

During motor imagery, we found differences in the laterality of ERD between the shoulder girdle movement and finger pinching. Bilateral ERD during motor imagery of elevation of the shoulder girdle and contralateral ERD during motor imagery of finger pinching were similar to those observed in actual elevation of the shoulder girdle or finger pinching (Figs. 2 and 3). Note that the ERD amplitude itself was not different between motor imagery and execution. A number of previous studies have reported that brain activity during imagery and execution of hand or foot movements are similar [50–52], and the present results suggest that the neural substrate for motor imagery of elevation of the shoulder girdle may be shared with motor execution.

A previous study reported that the ERD amplitude of actual motor execution was larger compared to that of motor imagery [53]. We argue that this difference may be due to a difference in the effector used or in the hemisphere where ERD was recorded. Nikulin and colleagues [53] used quick abductions of the thumb for the motor execution/imagery tasks to show larger ERD in motor execution than in imagery. In our experiments, we also observed larger ERD for finger motor execution than finger motor imagery, although it did not reach statistical significance. In contrast, shoulder motor execution and shoulder motor imagery, both of which are used to calculate ipsilateral ERD, showed almost no difference in our experiments. Thus, we assume that differences between the muscles involved in movements of the shoulder and finger, the difficulty of kinesthetic motor imagery of shoulder and finger movements, or the structure of the ipsilateral and contralateral hemispheres might result in conflicting results.

To further validate the speculation that the neural substrate for motor imagery of elevation of the shoulder girdle may be shared with motor execution, we tested the association of ERD during shoulder motor imagery with i-CST excitability, assessed by TMS over M1. The results showed that larger MEP amplitudes in the trapezius were associated with greater ERD in the ipsilateral hemisphere (Fig. 5). These results physiologically support the hypothesis that ipsilateral ERD reflects the excitability of uncrossed pathways projecting to the trapezius. Therefore, despite the fact that the neuroanatomical properties of the cortical projections to shoulder muscles are different from those to distal muscles [16–20] and that intraspinal circuitries also differ [21], we demonstrated that ERD in the ipsilateral hemisphere served as a biomarker for the functional contribution of the ipsilateral M1 to shoulder movements. This was similar to the relationship between contralateral ERD and finger movements. In the same experimental setup with the same participants, we also showed that larger MEP amplitudes in the FDI were associated with greater ERD in the contralateral hemisphere, which is consistent with the findings of a previous study [1]. This result validated our experimental paradigm for investigating the effects of ERD in M1 on CST excitability.

Correlation between MEP and ERD was unspecific and the number of participants showing the correlation is limited in the current experiments, even though it was significant (Fig. 5). However, EEG records sensor signals over the scalp and not cortical current sources. EEG is, therefore, noisy because of signal contamination from the environment. The EEG waveform is also skewed because of the complex geometric structure of the cortical surface insulated by cerebrospinal fluid and the skull, resulting in distortion of the frequency spectrum. Moreover, different physiological modalities far from the sensorimotor system, such as attentional or cognitive systems, also generate the same 8-13 Hz frequency signal. As such, methodological and physiological limitations exist with EEG, and ERD is not a perfect measure for showing corticospinal tract excitability. This might lead to the low to moderate correlations between MEP and ERD in the current study. Therefore, at the current time we must restrict our finding that if we can detect ERD stemming from motor-related neural activities, this will reflect cortical excitability.

The current results suggest that learned control of ipsilateral ERD with motor imagery-based BCI targeting elevation of the shoulder girdle might unmask i-CST function. Learning effects on i-CST coupled with the assessment of contralateral corticospinal tract excitability should be examined in the future. In post-stroke motor rehabilitation, increased ipsilateral cortical activity is known to be correlated with good recovery in axial muscles [13]. Therefore, our findings may have implications for selecting EEG channels in ERD-based BCI neurorehabilitation, aiming for upper-limb motor recovery. Specifically, in the future we may be able to improve shoulder motor function by enhancing ERD in the ipsilateral hemisphere by BCI, while improving finger motor function by enhancing ERD in the contralateral hemisphere. Such selective interventions may lead to better clinical outcome of upper-limb motor recovery in total.

## Conclusions

In the present study, we found that ERD increased in bilateral hemispheres during both execution and imagery of elevation of the shoulder girdle. Investigating i-CST excitability using single-pulse TMS over the primary motor cortex confirmed that the amplitude of ERD in the ipsilateral hemisphere was correlated with the MEP amplitude in the trapezius. These results suggest that ipsilateral EEG mu rhythm during motor imagery reflects the excitability of uncrossed pathways projecting to the trapezius, and this EEG feature may be used in BCI rehabilitation for functional recovery of proximal arm movements.

## Abbreviations

BCI: Brain-Computer Interface
c-CST: Contralateral corticospinal tract
CL: Central left
CR: Central right
EEG: Electroencephalography
EMG: Electromyography
ERD: Event-related desynchronization
FDI: First dorsal interosseous
FL: Front left
FR: Front right
i-CST: Ipsilateral corticospinal tract
LI: Laterality index
M1: Primary motor cortex
MEP: Motor-evoked potential
OL: Occipital left
OR: Occipital right
PL: Posterior left
PR: Posterior right
RMT: Resting motor threshold
SMR: Sensorimotor rhythm
TMS: Transcranial magnetic stimulation
